# Five-year follow-up of Japanese patients with Paget's disease of the bone after treatment with low-dose oral alendronate: a case series

**DOI:** 10.1186/1752-1947-4-166

**Published:** 2010-05-31

**Authors:** Kousuke Iba, Junichi Takada, Takuro Wada, Toshihiko Yamashita

**Affiliations:** 1 Department of Orthopedic Surgery, Sapporo Medical University School of Medicine, Chuo-ku, Sapporo, 060-8543, Japan; 2 Kitago Orthopedic Clinic, Kitago, Shiroishiku, Sapporo, 003-0833, Japan

## Abstract

**Introduction:**

Paget's disease of the bone is characterized by focal abnormalities of increased bone turnover affecting one or more sites throughout the skeleton. Although this disease is rare in Japan, it is common in western and southern Europe, and among British migrants in Australia and New Zealand. Bisphosphonates have been widely used for the treatment of Paget's disease of the bone and are considered to be the treatment of choice. However, there have been few reports on the long-term follow-up examination of patients after their treatment with bisphosphonates.

**Case presentation:**

We report the treatment with a low dose of oral alendronate (5 mg per day) which was effective in reducing bone turnover and pain over the five-year follow-up period in two Japanese patients, a 66-year-old man and a 68-year-old woman, with Paget's disease of the bone. Furthermore, in one patient, no clinical symptoms, such as bone pain or increases in serum total alkaline phosphatase and urinary N-terminal telopeptide of type I collagen as markers of bone turnover, were observed over the patient's five-year follow-up period.

**Conclusions:**

To the best of our knowledge, this is the first report of a long-term follow-up of patients with Paget's disease of the bone after a six-month treatment with low-dose oral alendronate (5 mg per day).

## Introduction

Paget's disease of the bone (PDB) is characterized by focal abnormalities of increased bone turnover affecting one or more sites throughout the skeleton [1]. Although this disease is rare in Japan [2,3], it is common in western and southern Europe, and among British migrants in Australia and New Zealand [4,5]. The rapid rate of bone turnover in PDB leads to structural abnormalities, reduced mechanical strength, and increased risk of pathological fractures among patients [1].

The main indication for the medical treatment of PDB is bone pain that is thought to be due to increased metabolic activity. Pagetic bone pain responds well to bisphosphonates that inhibit osteoclastic bone resorption [1,3,5,6]. Bisphosphonates have been widely used for PDB and are now considered to be the treatment of choice [1,6-8]. In contrast, only a few long-term studies over a period of more than five years have been performed to follow up patients with PDB after their treatment with bisphosphonates in Japan [7].

We previously reported that the administration of low-dose oral alendronate (5 mg per day) for six months was effective in reducing bone pain and bone turnover in two Japanese patients with PDB, although the follow-up period was only one year after the conclusion of alendronate treatment [9]. In this case report, we report the five-year follow-up of these patients. In both cases, treatment with low-dose oral alendronate was effective in reducing bone turnover and pain over the five-year follow-up period. Furthermore, in one patient, no clinical symptoms, such as bone pain or increases in serum total alkaline phosphatase (T-ALP) and urinary N-terminal telopeptide of type I collagen (uNTX) as markers of bone turnover, were observed over the five-year follow-up period. During this period, our patient also did not require any additional treatment. To the best of our knowledge, this is the first report of a long-term follow-up of patients with PDB after a six-month treatment with low-dose oral alendronate (5 mg per day).

## Case presentation

### Case 1

**Figure 1:**
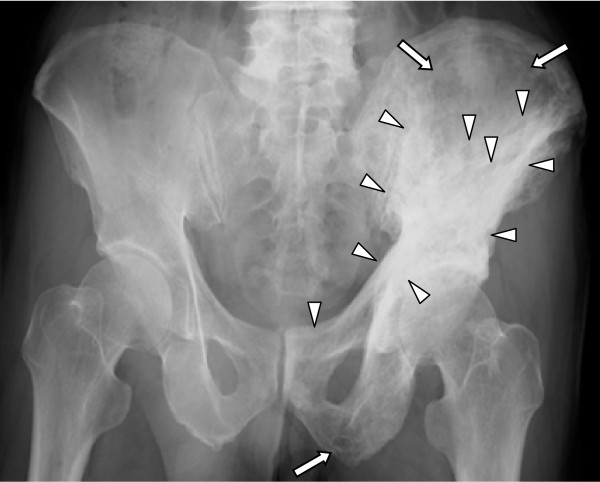
**Radiography of patient 1 showed pagetic changes as osteolytic (white arrow) and sclerotic (white arrow head) changes in the left acetabulum, half of the ilium, and the pubic bone**.

**Figure 2:**
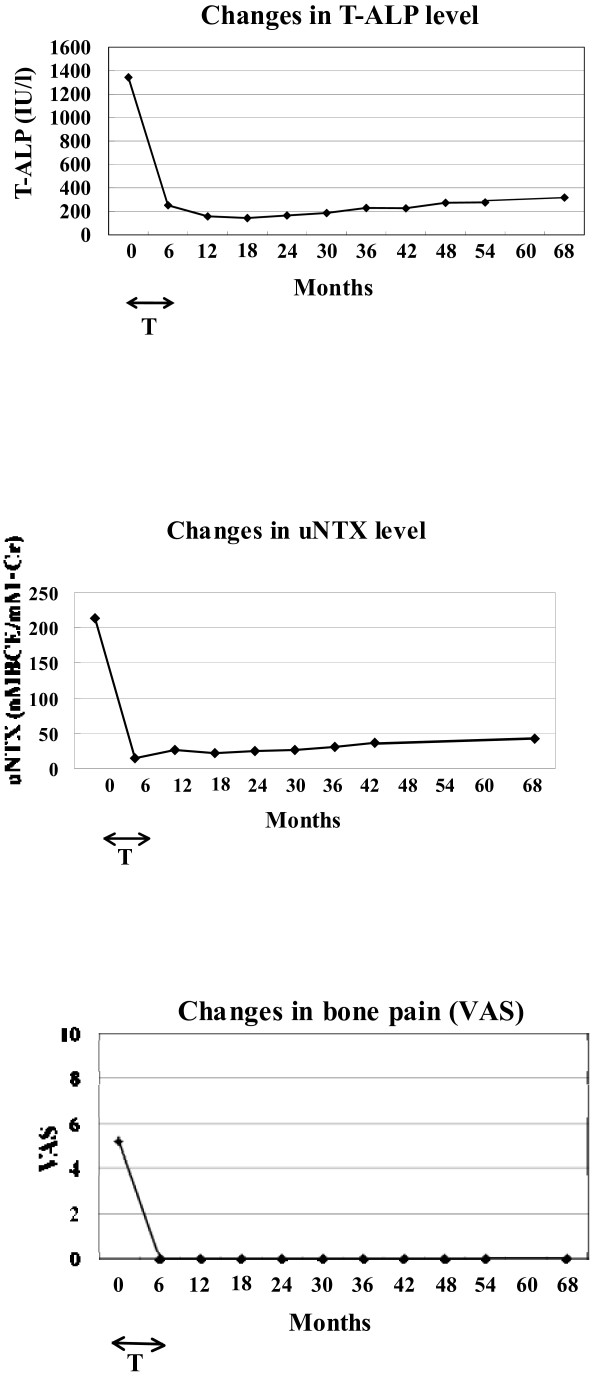
**Changes in T-ALP level, uNTX level, and bone pain (VAS) after treatment with a low dose of alendornate for six months (Patient 1)**. **(A) **T-ALP and **(B) **uNTX levels returned to a normal range, and **(C) **bone pain (VAS) disappeared within six months. These remissions continued for the five years since the discontinuation of the treatment. T-ALP (IU/L), serum total alkaline phosphatase; uNTX (nMBCE/mM·CR), urinary N-terminal telopeptide of type I collagen; VAS, visual analogue scale; T, duration of the treatment with alendronate (0 to 6 months).

A 66-year-old Japanese man presented with low back pain (visual analogue scale (VAS) = 5.2). There was no notable disease in his past medical records. Our patient's weight, height and body mass index (BMI) were 67.5 kg, 164.0 cm, and 25.1, respectively. Our patient was diagnosed with PDB based on typical features such as radiographic osteolytic and sclerotic changes in the pelvic bone (Figure 1), bone scintigraphy showing hot spots in the affected bones, and markedly increased levels of T-ALP (1344 IU/lL; normal range 110-370 IU/L) and uNTX (213 nmol bone collagen/mmol·creatinine [nMBCE/mM·Cr]; normal range 9.3 to 54.3). In addition, an open biopsy was performed to confirm the diagnosis. He was treated with oral alendronate at 5 mg per day, which led to the disappearance of his low back pain (VAS = 0) and the normalization of his T-ALP and uNTX levels within six months [9]. Since he was discontinued on his alendronate treatment, he has not complained of bone pain and his T-ALP and uNTX have remained at normal levels for the entire five-year follow-up, thus requiring no additional treatment (Figure 2).

### Case 2

**Figure 3:**
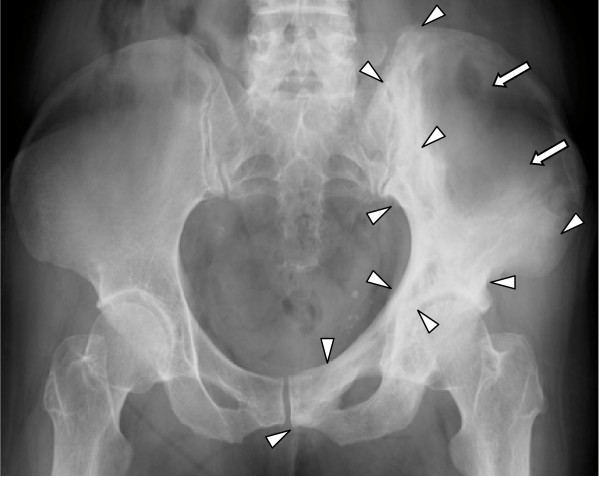
**Radiography of patient 2 showed pagetic changes as osteolytic (white arrow) and sclerotic (white arrowhead) changes in the left acetabulum, half of the ilium, and the pubic bone**.

A 68-year-old Japanese woman complained of low back pain and excessive warmth over her bones (VAS = 6.6). Nothing notable showed up in her past medical records. Her body weight, height and BMI were 56.1 kg, 155.2 cm and 23.3, respectively. Radiography showed typical Pagetic changes (Figure 3). A bone scintigraphy showed hot spots in her pelvic bone, and her T-ALP (593 IU/L) and uNTX (234.6 nMBCE/mM·Cr) levels were elevated as in the first patient. An open biopsy was performed to confirm diagnosis. After the oral administration of alendronate at 5 mg per day, her low back pain and excessive warmth over the bones disappeared (VAS = 0), and her T-ALP and uNTX levels returned to the normal range within six months [9]. After the six-month course of treatment, the normal levels of T-ALP and uNTX, as well as bone pain relief were maintained for one and a half years without any further treatment.

**Figure 4:**
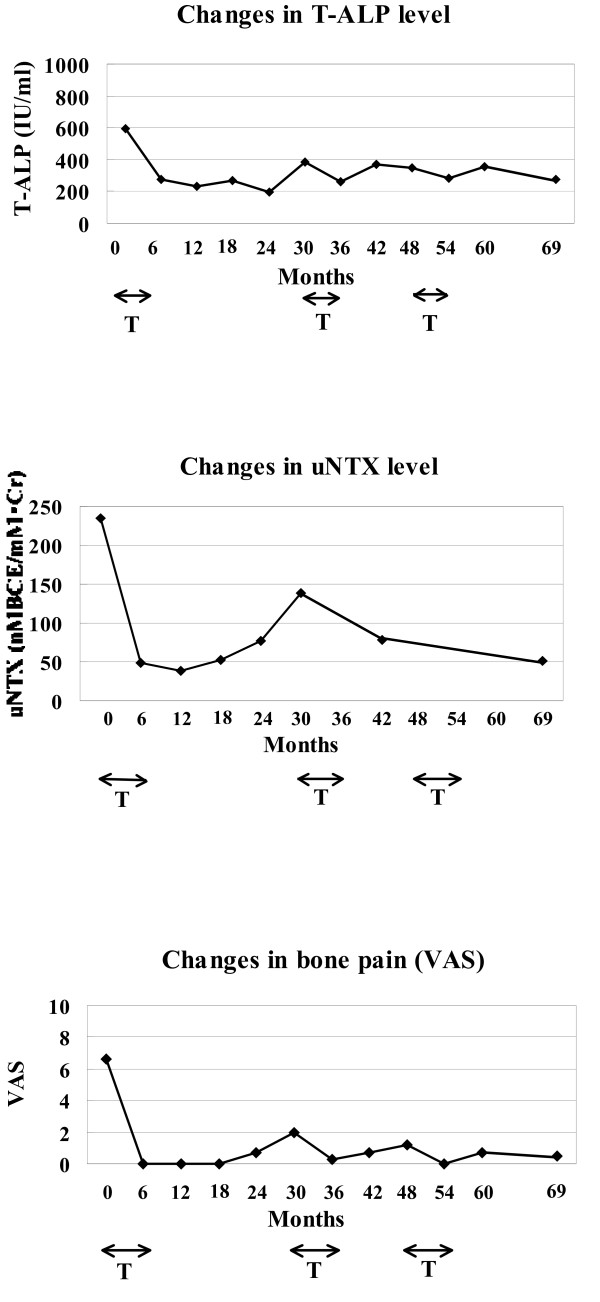
**Changes in T-ALP level, uNTX level, and bone pain (VAS) after the treatment with low-dose alendornate for six months (Patient 2)**. **(A) **T-ALP and **(B) **uNTX levels returned to normal range and, **(C) **bone pain (VAS) disappeared within six months. These remissions continued throughout the 1.5 to 2 years since the discontinuation of the treatment, although T-ALP and uNTX levels were slightly elevated above the normal range and was accompanied with mild bone pain. A resumption of alendronate treatment rapidly decreased the levels of **(A) **T-ALP and **(B) **uNTX, and **(C) **improved bone pain. T-ALP (IU/l), serum total alkaline phosphatase; uNTX (nMBCE/mM·CR), urinary N-terminal telopeptide of type I collagen; VAS, visual analogue scale; T, duration of the treatment with alendronate (0 to 6, 30 to 36, and 48 to 54 months.)

In contrast to our patient in Case 1, however, her T-ALP and uNTX levels again increased beyond the normal range, accompanied with mild bone pain at two years after the discontinuation of treatment. We resumed treatment with low-dose alendronate for six months. Her T-ALP and uNTX levels rapidly decreased and her bone pain improved within a few months, and this remission in T-ALP level, uNTX level and bone pain was maintained for more than one year. A year after the second course of treatment, her T-ALP again rose slightly, but the administration of low-dose alendronate was again successful in rapidly improving her T-ALP level (Figure 4).

## Discussion

Among bisphosphonates, orally administered etidronate, tiludronate, alendronate, and risedronate, as well as intravenous pamidronate and zoledronic acid have been widely used for the treatment of PDB [1,6]. Alendoronate and risedronate are particularly potent oral anti-resorptive drugs for the treatment of PDB, with effects that are comparable with those of intravenous pamidronate and zoledronic acid [1,6,8,10]. The most widely recommended protocols for the treatment of PDB with oral bisphosphonates is 40 mg/day of alendronate for six months and 30 mg/day of risedronate for two months [1,6].

About six years ago, we started treatment for two patients with PDB using low-dose oral alendronate (5 mg per day, orally) due to the following factors: (a) only etidronate was available for the treatment of PDB in Japan at that time, although risedronate has recently been approved by the Ministry of Health, Labor and Welfare for the treatment of PDB (17.5 mg per day, orally) in July 2008; (b) a comparative study had indicated that alendronate was more effective than etidronate for the treatment of PDB [11], and (c) alendronate seemed to be more suitable for the treatment of Japanese patients since the dose of alendronate used for the treatment of osteoporosis in Japanese patients (5 mg per day) was half of that used for the treatment of osteoporosis in Caucasians (10 mg per day). Thereafter, we reported that the therapeutic efficacy of this treatment was equivalent to that of a high dosage of alendronate for one year after the cessation of treatment [9]. Furthermore, in this study, we have shown that the administration of low-dose alendronate for six months has afforded an effective treatment for PDB throughout the five-year follow-up.

Recently, several studies have indicated the possibility that long-term or high-dose bisphosphonate therapy could cause severe side effects, including osteonecrosis of the jaws [12,13], or lead to severe suppression of bone turnover (SSBT) [14]. For this reason, the administration of low-dose alendronate might be an option for the medical management of patients with PDB, although it remains unknown whether most Japanese and Caucasian patients would respond well to this treatment.

A diagnosis of PBD can usually be confirmed from the presence of clinical symptoms such as pain in the involved bone, typical plain radiological features of osteosclerosis alternating with areas of osteolysis, a radionuclide bone scan indicating markedly increased tracer uptake in the affected bone, and the elevation of serum level of alkaline phosphatase [1-3]. Although both of our patients had the typical features described above, open biopsies were also performed. As PDB is uncommon in Japanese compared to among Caucasians, bone biopsy was recommended to differentiate PDB from secondary tumors such as metastases from prostate cancer or breast cancer, and other sclerosing bone dysplasia [2,3].

To monitor disease activity and the effects of treatment, we measured T-ALP and uNTX levels of our patients every three to six months and assessed their bone pain as described in previous studies [1,3,11,15]. In both of our patients, T-ALP and uNTX reached a normal level and the bone pain and excessive warmth over the bones disappeared within six months from the start of alendronate treatment [9]. Interestingly, the progress of our patients during the five-year follow-up after the discontinuation of treatment differed. In one patient (Case 1), his T-ALP level remained within the normal range and no bone pain was reported throughout the five-year follow-up, thus requiring no additional treatment. In contrast, our second patient (Case 2) was re-treated twice (Figure 2) for her increased T-ALP level. However, the additional treatment with low-dose alendronate rapidly normalized her T-ALP level within a few months and she was able to maintain this level for more than one year without requiring medication. Thus, each patient with PDB responded well to the bisphosphonate therapy for at least one year since the treatment was discontinued. These results indicate that the appropriate length of bisphosphonate treatment for PDB, as well as the length of follow-up, remains unclear.

The recommended regimens of oral bisphoshonate therapies for PDB in various parts of the world are based on high-dose administration for two to six months [1,3,6]. In our reports based on a five-year follow-up, low-dose alendronate afforded an effective therapy for two Japanese patients with PBD. In Case 2, we had to resume her treatment twice to maintain normal levels of T-ALP and uNTX, and the relief of bone pain. These results suggested that the effects of low-dose oral alendronate might have some limitations for the treatment of PDB.

Guidelines for the diagnosis and management of PDB has recently been reported in Japan [3], which indicate that risedronate (17.5 mg per day orally) has been available to treat Japanese patients with PBD since July 2008. It might be better to treat patients like Case 2 with a high dose of oral risedronate (17.5 mg per day) for eight weeks in accordance with the guidelines for the management of PDB in Japan [3].

## Conclusions

In this report, we have shown that the administration of low-dose alendronate (5 mg per day) for six months has afforded an effective treatment for PDB over the five-year follow-up in two Japanese patients with Paget's disease of the bone. To the best of our knowledge, this is the first report of a long-term follow-up of patients with PDB after a six-month treatment with low-dose oral alendronate.

## Consent

Written informed consent was obtained from our patients for publication of this case report and any accompanying images. A copy of the written consent is available for review by the Editor-in-Chief of this journal.

## Competing interests

The authors declare that they have no competing interests.

## Authors' contributions

KI and JT treated our patients with Paget's disease of the bone by using low-dose oral alendronate (5 mg per day) and observed them over the five-year follow-up period. KI, JT, TW and TY analyzed and interpreted our patient data and the treatment. KI was a major contributor in writing the manuscript. All authors read and approved the final manuscript.
